# Gene Expression and Carcass Traits Are Different between Different Quality Grade Groups in Red-Faced Hereford Steers

**DOI:** 10.3390/ani11071910

**Published:** 2021-06-27

**Authors:** Bailey Engle, Molly Masters, Jane Ann Boles, Jennifer Thomson

**Affiliations:** 1 Centre for Animal Science, Queensland Alliance for Agriculture and Food Innovation, St. Lucia, QLD 4072, Australia; b.engle@uq.edu.au; 2 Department of Animal and Range Sciences, Montana State University, Bozeman, MT 97071, USA; molly.masters@montana.edu (M.M.); jboles@montana.edu (J.A.B.)

**Keywords:** gene expression, quality grade, beef cattle

## Abstract

**Simple Summary:**

Producing a consistent and positive experience for beef consumers is challenging. The gene expression in muscle at harvest may provide insight into better prediction of United States Department of Agriculture (USDA) quality grade. In this pilot study muscle samples were collected at harvest on sixteen steers with a similar background and identical management. Muscle transcripts were sequenced to determine gene expression. Transcripts related to the extracellular matrix, stem cell differentiation, and focal cell adhesions were differentially expressed in muscle tissue from carcasses with differing USDA quality grades. This confirmed the application of this technique to provide insight into muscle development and fat deposition necessary for better prediction and selection to improve consistency and consumer experience.

**Abstract:**

Fat deposition is important to carcass value and some palatability characteristics. Carcasses with higher USDA quality grades produce more value for producers and processors in the US system and are more likely to have greater eating satisfaction. Using genomics to identify genes impacting marbling deposition provides insight into muscle biochemistry that may lead to ways to better predict fat deposition, especially marbling and thus quality grade. Hereford steers (16) were managed the same from birth through harvest after 270 days on feed. Samples were obtained for tenderness and transcriptome profiling. As expected, steaks from Choice carcasses had a lower shear force value than steaks from Select carcasses; however, steaks from Standard carcasses were not different from steaks from Choice carcasses. A significant number of differentially expressed (DE) genes was observed in the longissimus lumborum between Choice and Standard carcass RNA pools (1257 genes, *p* < 0.05), but not many DE genes were observed between Choice and Select RNA pools. Exploratory analysis of global muscle tissue transcriptome from Standard and Choice carcasses provided insight into muscle biochemistry, specifically the upregulation of extracellular matrix development and focal adhesion pathways and the downregulation of RNA processing and metabolism in Choice versus Standard. Additional research is needed to explore the function and timing of gene expression changes.

## 1. Introduction

Subcutaneous fat thickness and marbling (intramuscular fat) are integral parts of the United States grading systems and are a large portion of the pricing system. Many certification systems, such as Certified Angus Beef™, are utilizing marbling to reduce variation in tenderness. These schemes are being developed because inconsistency of the tenderness in meat is a major concern in the beef industry due to reduced consumer acceptance [1]. Many researchers have indicated steaks from Prime and Choice carcasses were more tender than steaks from Select carcasses [2,3,4]. Consequently, providing consistent tender products has become a major concern for beef cattle producers. 

Subcutaneous fat is a major factor in the prediction of yield from a carcass, while marbling has been used to try and guarantee tenderness [5]. As time on feed increases, marbling and subcutaneous fat increase, leading to an increase in both the amount of subcutaneous fat and the number of carcasses grading USDA Choice [6]. Techniques that would allow us to better understand how fat deposition is controlled at the transcript level would help to reduce “overfeeding” and improve the efficiency of grain-fed beef production. Ongoing research is required in order to gain a better understanding of the genetic expression of these traits and how selection plays a role in the transmission and expression of these economically significant characteristics The development of high-throughput transcriptomic analysis has provided a powerful tool to study gene expression by allowing simultaneous measurement of the entire transcriptome [7,8,9], including mRNA, transcription factors, and gene-splicing variants that can impact gene activity. Combined with the traditional tools of physiology and biochemistry, a transcriptomic level of understanding will progress our knowledge of cell biology and bovine muscle biochemistry. Previous studies have evaluated gene expression in animals with varying degrees of fatness or intramuscular fat [10,11,12]. Many of these studies have focused on small sets of genes with a known function such as peroxisome proliferator-activated receptors (PPARs) and sterol regulatory element-binding proteins (SREBPs) [11,12]. The specific objective of this pilot study was to evaluate the relationship between USDA meat quality grade and gene expression to look for additional genes, pathways, and networks that relate to meat quality grade. This exploratory research provides an overview of gene expression changes in relation to quality grade within a cohort with a common genetic background, management, age, and days on feed.

## 2. Materials and Methods

Sixteen red-faced Hereford steers were fed at Fort Keogh Agricultural Research Station under the standard feedlot Standard Operating Procedures (SOP). These animals were genetically similar and were born within a three-week period and handled from birth through harvest as the same group with a common diet and management. All animals received care according to the “Guide for the Care and Use of Agricultural Animals in Agricultural Research and Teaching, 3rd Edition” and under the supervision of the Fort Keogh Animal Care and Use Committee (ACUC No.: 020104-9). Animals within the same cohort that produced carcasses grading Standard, Select, and Choice at the same age and days on feed were sampled for this study. 

The animals were harvested after all steers had been fed in the same pen for 270 days in the feedlot. Animals were harvested at a federally inspected facility. At harvest, a muscle sample from the longissimus lumborum (LL) near the posterior end was collected using a Tru-cut biopsy needle [13], snap-frozen in liquid nitrogen, and stored for subsequent RNA extraction within 30 min following exsanguination. Carcass data were collected 24 h postmortem to calculate yield grade and determine quality grade following USDA guidelines [14]. Carcasses were ribbed between the 12th and 13th rib exposing the longissimus dorsi. Marbling determination was carried out a minimum of 20 min after ribbing to give time for the muscle to oxygenate. Carcass characteristics were measured by an individual with 25 years of experience in carcass data collection and the training of people to collect carcass data. USDA marbling standards were used to ensure the consistency of marbling scores. Physiological maturity of the carcass was assessed by evaluating the ossification of the bones in the vertebral column and evaluating lean color and the color and shape of the rib bones. Fat thickness was measured at the 12th rib at a distance ¾ of the way from the spinal column towards the ventral side of the carcass with a metal ruler divided into 0.1-inch increments. The measurements were later converted to cm for statistical analysis. The rib eye area was measured using a standard grid that had 10 dots per square inch, and dots surrounded by lean were the ones counted. The square inches measured with the grid were converted to square centimeters for statistical analysis. Internal fat was estimated as a percentage of the carcass weight. After 24 h, striploins (North American Meat Purveyors—NAMP 180) were removed from the left side of each carcass. These striploins were cut into 2.54 cm steaks, vacuum packaged, and aged for 1, 3, 7, 14, and 21 days. These samples were used to determine shear force values for each steak. 

The biopsy samples collected for RNA-seq analysis averaged 165 mg of tissue. A Qiagen RNeasy spin-column kit was used for RNA extraction. Extracted RNA samples had an average concentration of 35.88 ng/uL. RNA quality was assessed using an Agilent 2200 Tapestation and RNA Screentape. The RIN (RNA integrity) values averaged 7.06 with a minimum value of 6.7. The extracted RNA was depleted of ribosomal RNA using an Invitrogen Ribominus kit and then used to create individual cDNA libraries that were then randomly allocated to one of two pools for each quality grade of Standard, Select, and Choice. These pools were created to provide a biological replicate sample and to focus our pilot analysis on genes that were differentially expressed by quality grade and not due to individual animal variation. These pools were then sequenced on an Ion Proton next-generation sequencing platform according to the manufacturer’s instructions. The sequencing reads generated were aligned to the known bovine consensus sequence (Btau 4.2), and a normalized count of reads was generated to determine the expression of each known gene and gene isoforms using Strand NGS RNA-seq software (version 2.8, Avadis, Bangalore, India). Differentially expressed genes and transcripts were calculated using a pooled z-test with a test statistic of

$$Z=\frac{\sqrt{\frac{x_{1}}{N_{1}}}-\sqrt{\frac{x_{2}}{N_{2}}}}{\sqrt{\frac{1}{4N_{1}}+\frac{1}{4N_{2}}}}$$

$$x^{1}=\sum_{i=1}^{q_{1}}x_{1,}x_{2}=\sum_{i=1}^{q_{2}}x_{2i},N_{1}=\sum_{i=1}^{q_{1}}N_{1i}\;and\;N_{2}=\sum_{i=1}^{q1}N_{2i}\;\;\;$$

and with *p*-values calculated by

if Z<0, p=2*Φ(Z)

$$\begin{array}{c} \mathrm{else}\;p=2*\left(1-\Phi \left(Z\right)\right)\;\\ \mathrm{where}\;x_{1}\text{:} \end{array}$$

A Bonferroni correction was used to account for multiple comparisons and determine a corrected *p*-value. Additionally, fold change between Standard and Choice, as well as up- and downregulation, was determined for a Standard vs. Choice comparison. Gene set enrichment analysis and network and pathway analysis were used to identify genes and gene networks that relate to growth rate, tenderness, and carcass quality. Functional analysis was performed using DAVID bioinformatics software (version 6.8, Frederick National Laboratory, Frederick MD, USA) [15]. Library preparation and bioinformatics analysis were conducted according to the SOP and pipeline developed at Colorado State University Infectious Disease Unit and published in Das et al. (2016) [16,17]. RNA-seq data can be accessed at 10.5281/zenodo.4993828.

Shear force was determined using previously published methods [18]. Briefly, steaks were thawed at 4 °C for 24 h, weighed, and two copper constantan thermocouples (Omega Engineering, Stanford, CT, USA) were inserted into the geometric center of each steak and placed under (10 cm from element) an electric broiler and cooked to a final internal temperature of 70 °C, turning when the samples reached 35 °C. Each steak was weighed before and after cooking to determine cook loss. Eight to ten square samples (1.27 cm × 1.27 cm × 2.54 cm) for shear force evaluation were removed parallel to the fiber direction from each steak that was cooked and cooled. Samples were sheared once perpendicular to the fiber direction with a TMS 30 Food Texturometer fitted with a Warner–Bratzler shear attachment. The average of the samples sheared was used for statistical analysis in relation to the differentially expressed genes and transcripts.

Individual animals were used as the experimental unit (*n* = 16; 6—Choice, 5—Select, 5—Standard). The Generalized Linear Model (GLM) procedure of SAS, Statistical Analysis System (Cary, NC, USA) was used to analyze carcass and tenderness data. Lease Square Means (LSMEANS) procedures of SAS were employed due to an unequal number of observations upon which to compare differences between variables. Interaction between aging time and treatment was tested. It was not significant, so the data were analyzed as independent variables.

## 3. Results and Discussion

There were significant differences in carcass characteristics between carcasses of different quality grades. As expected, marbling scores were significantly lower for Standard and Select carcasses. The hot carcass weight (HCW) was significantly lower for Standard carcasses but was not different between Choice and Select carcasses. Ribeye area (REA) and kidney, pelvic, and heart fat percentage (KPH) were also significantly different between all three grades, with Choice having the largest REA and highest KPH and Standard the lowest (Table 1). Fat thickness, however, was not different between Choice and Select carcasses, while the Standard carcasses had less subcutaneous fat. Other researchers have reported relationships between larger carcasses and REA, as well as increased fat with larger carcass weights from concentrate-fed animals [6,19,20]. Fat deposition is a balance between energy consumed versus energy used. The steers were all of a very similar genetic background and chronological age and had been managed in a similar manner throughout their lifespan. Therefore, steers resulting in Standard-grade carcasses with a smaller REA and higher variability in REA suggests they had not completed muscle growth and were therefore using more energy for continued muscle growth and storing less fat [21,22,23]. Furthermore, Select and Choice carcasses had significantly higher yield grades than Standard carcasses, suggesting a lower percentage of closely trimmed retail cuts could be achieved from those carcasses.

There was no significant interaction between carcass grade and days of aging for shear force. Therefore, main effect means are presented in Table 1 and Table 2. Steaks from Standard and Choice carcasses had significantly lower average shear force values than steaks from Select carcasses (Table 1). Obuz et al. [3] also reported steaks from Select carcass being less tender than steaks from Choice carcasses, while Legako and coworkers [24] reported no difference in sensory tenderness scores for longissimus lumborum samples from different quality grades. Unfortunately, there is much less research on the tenderness of steaks from Standard-quality-grade carcasses, so there are much less data to compare these results with. In contrast, Smith et al. [25] and Tatum et al. [6] reported that sensory panel ratings increased, and shear force values decreased as the marbling score and fat thickness increased. The variation in reported tenderness information suggests that other factors are affecting tenderness along with the changes in intramuscular and subcutaneous fat. As expected, postmortem aging resulted in a decrease in shear value (Table 2) along with a reduction in variation within a sample [26,27,28]. The data reported here for shear force value are similar to other data reported when square cores were used to determine shear force [18,19,29]. 

In this exploratory study, there were a significant number of differentially expressed (DE) genes (1257 genes, *p* < 0.05) in LL muscle tissue from Choice and Standard carcasses and a similar number (1249 genes, *p* < 0.05) between LL muscle tissue from Choice and Select carcasses. There were also a significant number of DE genes (2376 genes, *p* < 0.05) between LL muscle from Select and Standard carcasses. The complete list of all DE genes with *p* ≤ 0.01 is attached as Supplementary Materials Tables S1–S3. The complete list of DE genes with two-fold or greater changes is shown in the attached Supplementary Materials Tables S1–S3. A subset of DE genes with high fold changes (>2-fold change, *p* ≤ 0.01) either up- or downregulated in Choice versus Standard quality grades is shown in Table 3 and Table 4, respectively. A subset of DE genes with high fold changes (>2-fold change, *p* ≤ 0.01) either up- or downregulated in Choice versus Select quality grades is shown in Table 5 and Table 6, respectively. Upregulated DE genes observed in samples from Standard versus Choice carcasses function in transcriptional regulation, inter- and extracellular signaling, growth, muscle metabolism, and angiogenesis (Table 3). Differentially expressed genes that were downregulated function in transcriptional regulation, amino acid transport, and metabolism (Table 4). Upregulated DE genes observed in samples from Select versus Choice carcasses function in extracellular and cytokine signaling, catalytic activity, protein binding, and positive regulation of cell migration and angiogenesis (Table 5). Differentially expressed genes that were downregulated function in anti-inflammatory activity, lipoprotein metabolism, immune response, T-cell activity, and protein catabolism (Table 4). Some of the observations are similar to those previously reported by Clark et al. [30], who examined muscle gene expression associated with increased marbling in beef cattle. Clark et al. [30] reported a 1.38-fold increase in G-protein-coupled receptor 153 (GPR153), a 1.41-fold change in the Matrix-remodeling-associated 8 [29] transcript, and a 1.88-fold increase in Pleckstrin and Sec 7 domain-containing (PSD) transcripts when comparing high-marbled vs. low-marbled beef cattle. This study found similar results, with a 3.7-fold change in GPR153 and 3.08-fold change in MXRA8 and a 2.43-fold decrease in PSD, respectively, in muscle from Choice versus Standard carcasses (Supplementary Materials Table S2). Roberts et al. [31] utilized a targeted quantitative real-time PCR approach to examine gene expression in muscle. They found an initial decrease and then an increase in the expression of NADH dehydrogenase 2 (ND2) when examining muscle with no intramuscular fat transitioning to mature intramuscular fat. This study had a 7.42-fold increase in the expression of ND2 in the comparison of Standard vs. Choice muscle tissue. Similarly, Roberts et al. [31] showed a high level of expression of tissue metallopeptidase inhibitor 4 (TIMP4) in muscle early in the development of intramuscular fat with a decrease and later increased as intramuscular fat developed. This study showed a 2.6-fold increase in TIMP4 in muscle from Standard carcasses compared to Choice muscle tissue (Supplementary Materials Table S2). Lastly, Fonseca et al. [9] showed a 0.77-fold decrease in the expression of heme oxygenase 1 (HMOX1) with increasing tenderness in Nellore cattle. This study observed a similar 2-fold decrease in HMOX 1 when comparing Standard vs. Choice muscle tissue with no associated difference in tenderness.

Functional analysis was run using DAVID (6.8) bioinformatics software, which revealed differences in the underlying pathways regulating muscle cell growth and proliferation. The complete list of enriched pathways and processes is shown in Supplementary Materials Table S3. A subset of significantly (*p* < 0.1) enriched KEGG pathways and gene ontology terms, including cellular components, biological processes, and molecular functions, is shown in Table 7 and Table 8, respectively. Biological processes of upregulated genes are associated with signaling pathways associated with inflammation, growth, and metabolism. Furthermore, upregulated genes associated with the extracellular matrix, stem cell differentiation, and focal adhesion were observed. Biological processes of downregulated genes are associated with RNA transport, degradation, and processing of RNA along with cellular metabolism. 

Many researchers have reported that MyoD, myogenin, Myf5, and MURF 4 [32,33], along with the upregulation of the mTOR pathway [33], are necessary for increased muscle mass. Goodman et al. [34] stated that mTOR was necessary for up to 50% of the basal rates of protein synthesis. However, in this preliminary study, no increase in any of these signals was seen in muscle from carcasses that resulted in a Choice quality grade compared to Standard. One possible explanation is that the downregulation of RNA degradation or the modification of signals within the pathway leads to the stabilization of the RNA needed for the increased synthesis of muscle proteins, so there is no need to increase signaling to make more RNA. Another possible explanation is that the control of muscle growth is no longer under transcriptional control. This has been reported previously by Tao et al. [35], who reported a higher correlation between adipose tissue gene expression clusters and carcass traits than what they saw for muscle tissue gene expression [36]. Additionally, a recent review by Raza et al. [36] showed that microRNAs played a large role in the regulation of muscle growth during a similar period.

An increase in the genes associated with the extracellular matrix, stem cell differentiation, and focal adhesions would suggest there is still muscle growth occurring. The extracellular matrix is an important part of the development of muscle cells. Focal adhesions are needed to anchor the myofibrillar structure to connective tissue in the muscle to help translate the mechanical action. Flück et al. [37] reported that focal adhesion kinase (FAK) is associated with focal adhesions and is a key regulator of the focal adhesion complex. The upregulation of pathways associated with focal adhesion may result in increased FAK. Crossland and coworkers [38] reported that FAK is a key component of IGF-1 modulation in cell culture and postulated that it is a key player in muscle growth in humans. Additionally, Keady et al. [39] found that sire breed and genetic merit for carcass weight impacted the transcriptional regulation of the somatotropic axis in the muscle tissue of crossbred steers, including a greater gene expression of IGF-1 and a reduced transcript abundance of IGFBP3 in increased muscle growth potential.

The downregulation of amino acid transport and metabolism could indicate that the muscle in the Choice carcass had reached maximum muscle growth and was in the process of moving consumed nutrients into storage as opposed to using them for growth. Energy consumed that is greater than that needed for growth or maintenance will be stored as fat.

## 4. Conclusions

As expected, steaks from Choice carcasses had a lower shear value than steaks from Select carcasses; however, there were no differences in differentially expressed genes that could be related to tenderness. Upregulated DE genes observed in samples from Standard versus Choice carcass functions were related to transcriptional regulation, inter- and extracellular signaling, growth, muscle metabolism, and angiogenesis, while downregulated DE genes were related to transcriptional regulation, amino acid transport, and metabolism. Enriched processes, including extracellular matrix, stem cell differentiation, and focal adhesions, suggest continued muscle growth in addition to fat deposition in carcasses of a higher quality grade. This explorative analysis of muscle transcript expression along with the findings of others [35,36] may indicate that muscle tissue during the finishing phase is not primarily under transcriptional regulation and that the transcriptional of adipose tissue may have a more predominant role in the determination of carcass quality grade. Exploratory analysis of global muscle tissue transcriptome in Standard and Choice carcasses provides insight into muscle biochemistry, and while additional research is needed to explore the function and timing of gene expression changes, it appears to be a useful approach to providing insight and generating additional research questions and direction to improve understanding of muscle development and fat deposition.

## Figures and Tables

**Table 1:** Main effect of quality grade on average shear force and carcass characteristics of red-faced Hereford steers.

Grade	Shear Force (N ± SD)	HCW (k ± SDg)	Fat (cm ± SD)	REA (cm^2^ ± SD)	KPH (% ± SD)	Yield Grade (±SD)	Marbling ^1^ (±SD)
Standard	66.3 ^b^ ± 12.4	250.6 ^b^ ± 33.0	0.43 ^b^ ± 0.21	57.68 ^c^ ± 5.27	1.60 ^c^ ± 0.22	2.46 ^ab^ ± 0.4	266 ^c^ ± 16.7
Select	84.3 ^a^ ± 13.9	282.9 ^a^ ± 23.0	0.64 ^a^ ± 0.21	67.23 ^b^ ± 2.72	1.80 ^b^ ± 2.27	2.62 ^a^ ± 0.37	330 ^b^ ± 18.7
Choice	71.3 ^b^ ± 13.0	283.2 ^a^ ± 15.9	0.58 ^a^ ± 0.23	72.13 ^a^ ± 4.87	2.08 ^a^ ± 0.38	2.35 ^b^ ± 0.24	463 ^a^ ± 15.0
*p* value	<0.0001	<0.0001	0.0104	<0.0001	<0.0001	0.0077	<0.0001

^1^ 200 = Traces, 300 = Slight, 400 = Small, 500 = Modest. ^a, b, c^ means within the column with different superscripts are significantly different *p* ≤ 0.05.

**Table 2:** Main effect of aging on the shear force of steaks from red-faced Hereford steers.

Age (days)	Shear Force (N ± SD)
1	89.9 ^a^ ± 16.5
3	80.7 ^b^ ± 17.8
7	71.0 ^b^ ± 13.7
14	65.3 ^b^ ± 11.9
21	62.9 ^c^ ±9.7

^a, b, c^ Means within the column with different superscripts are significantly different *p* ≤ 0.05.

**Table 3:** Upregulated differentially expressed genes with high fold changes in Choice compared to Standard LD muscle.

Gene ID	Gene Name	Fold Change	Corrected *p*-Value	Function
TET1	Tet methylcytosine dioxygenase 1	12.37	0.031	DNA and iron binding
ETV5	ETS variant 5	9.89	0.045	Regulation of transcription, neuromuscular transmission
BRB	Brain ribonuclease	9.89	0.002	Nucleic acid binding, endonuclease activity, hydrolase activity
DKK3	Dickkopf WNT signaling pathway inhibitor 3	9.89	0.002	Multicellular organism development, negative regulation of WNT signaling
BASE2	Beta-secretase 2	9.28	0.003	Proteolysis, protein catabolic processes
TNFAIP2	TNF-alpha-induced protein 2	8.67	0.008	SNARE binding, exocytosis
HPGD	5-hydroxyprostaglandin dehydrogenase	6.80	0.016	Growth factor receptor signaling
PCGF6	Polycomb group ring finger 6	6.18	0.025	Negative regulation of transcription
SERPINE1	Serpin family E member 1	4.93	0.006	Angiogenesis, protease binding, enzyme inhibitor activity
ANKRD2	Ankyrin repeat domain 2	2.06	0.016	Protein/titin binding, skeletal muscle development/differentiation

**Table 4:** Downregulated differentially expressed genes with high fold changes in Choice compared to Standard LD muscle.

Gene ID	Gene Name	Fold Change	Corrected *p*-Value	Function
RFC2	Replication factor C subunit	11.77	0.0008	DNA binding, DNA replication, Cell cycle activity
PQLC2	PQ loop repeat containing 2	9.01	0.0003	Amino acid transmembrane transport
PPM1F	Protein phosphatase, Mg^2+^/Mn^2+^ dependent 1F	8.09	0.0008	Protein dephosphorylation, positive regulation of transcription, negative regulation of protein kinase
TBPL1	TATA-box binding protein-like 1	4.56	0.027	DNA binding, positive regulation of transcription
GLB1	Galactosidase beta 1	4.31	0.028	Carbohydrate metabolic process, hydrolase activity
INSIG2	Insulin-induced gene	3.9	0.027	Lipid metabolic process
GALNT11	Polypeptide N-acetylgalactosaminyltransferase 11	3.74	0.027	Notch binding, protein glycosylation, transferase activity
TAF7-201	TATA-box binding protein associated factor 7	3.07	0.0004	Negative regulation of transcription, thyroid hormone signaling
NKTR	Natural killer cell triggering receptor	2.75	0.0005	Protein folding and protein refolding
PSMA4	Proteasome subunit alpha 4	2.17	0.0008	Proteolysis, protein catabolic processes

**Table 5:** Upregulated differentially expressed genes with high fold changes in Choice compared to Select LD muscle.

Gene ID	Gene Name	Fold Change	Corrected *p*-Value	Function
MX1	MX1 MX dynamin-like GTPase 1	11.25	<0.000	Extracellular signaling, immune response, recognition of pregnancy
CHRND	Cholinergic receptor nicotinic delta subunit	7.59	0.002	Muscle acetylcholine receptor
UBA7	Brain ribonuclease	7.24	<0.000	Cytokine signaling
RSAD2	Radical S-adenosyl methionine domain-containing 2	6.9	<0.001	Iron and sulfur binding, catalytic activity, protein self-association
OAS1Z	2′,5′-oligoadenylate synthetase 1	6.12	<0.001	Cytokine signaling, neutrophil gene expression
NUDT8	Nudix hydrolase 8	5.55	0.004	Coenzyme A diphosphatase
NMRAL1	NmrA-like redox sensor 1	5.52	<0.001	Protein binding, nuclear signaling
CELSR1	Cadherin EGF LAG seven-pass G-type receptor 1	5.42	0.005	Positive regulation of cell migration and angiogenesis
SQLE	Squalene epoxidase	5.23	0.01	Modulator of plasma membrane lipid profile, cholesterol synthesis inhibition
BOLA-DOB	Major histocompatibility complex, class II, DO beta	5.2	0.044	Immune function, self-recognition,

**Table 6:** Downregulated differentially expressed genes with high fold changes in Choice compared to Select LD muscle.

Gene ID	Gene Name	Fold Change	Corrected *p*-Value	Function
PLA2G7	Phospholipase A2 group VII	7.00		Anti-inflammatory activity, lipoprotein metabolism
BACH2	BTB domain and CNC homolog 2	6.52	0.01	Innate immune response, PAX5 signaling
ALK	ALK receptor tyrosine kinase	6.28	0.027	Cellular signaling
ZNF184	Zinc finger protein 184	6.02	0.001	Cellular signaling
ZP2	Zona pellucida glycoprotein 2	5.71	0.01	Reproduction, Ca2+ transport
SMOX	Spermine oxidase	5.67	0.02	ROS mediation, catabolism of polyamines
PRDM15	PR/SET domain 15	5.05	0.05	Neurogenesis, epigenetic modifier
TNFRSF12A	TNF receptor superfamily member 12A	4.86	0.006	Cytokine signaling
CD8A	CD8a molecule	4.61	0.016	T-cell activity, immune function
DNAJB14	DNAJ heat-shock protein family (Hsp40) member B14	4.53	0.001	Endoplasmic reticulum function, protein catabolism

**Table 7:** Gene set enrichment analysis of KEGG Pathways, and Gene Ontology of DE genes upregulated in Choice compared to Standard LD muscle.

KEGG Pathways		
Pathway Name	Count	*p*-Value
TNF signaling pathway	15	<0.001
Signaling pathway regulating pluripotency of stem cells	16	0.004
Thyroid hormone signaling pathway	14	0.004
Neurotrophin signaling pathway	14	0.009
Apoptosis	8	0.01
Insulin resistance	12	0.02
Type 2 diabetes mellitus	7	0.03
FoxO signaling pathway	13	0.03
Focal adhesion	17	0.05
Gene Ontology		
Cellular Compartment		
Proteinaceous extracellular matrix	27	<0.001
Cortical actin cytoskeleton	8	0.01
Biological process		
Canonical Wnt signaling pathway	13	<0.01
Negative regulation of catalytic activity	7	0.03
Regulation of cell migration	17	0.05
Molecular Function		
Endopeptidase activity	7	0.03
Metal ion binding	90	0.06

**Table 8:** Gene set enrichment analysis of KEGG Pathways and Gene Ontology of DE genes downregulated in Choice compared to Standard LD muscle.

KEGG Pathways		
Pathway Name	Count	*p*-Value
RNA transport	24	<0.001
RNA Degradation	25	<0.001
Spliceosome	19	0.002
cGMP-PKG signaling pathway	20	0.007
NF-kappa B signaling pathway	13	0.01
Lysosome	16	0.02
Basal transcription factors	8	0.02
Metabolic pathways	98	0.02
Vascular smooth muscle contraction	14	0.04
Gene Ontology		
Cellular Compartment		
Cytoplasm	288	<0.001
Small-subunit processome	8	0.009
Biological Process		
RNA methylation	4	0.002
Positive regulation of interferon-beta production	7	0.003
DNA repair	19	0.003
mRNA splicing	16	0.003
Negative regulation of MAP kinase activity	8	0.004
Molecular Function		
Metal ion binding	137	<0.001
Nucleic acid binding	58	<0.001
RNA methyltransferase activity	5	<0.001

## Data Availability

RNA-seq data can be accessed at 10.5281/zenodo.4993828.

## Supplementary Materials

The following are available online at https://www.mdpi.com/article/10.3390/ani11071910/s1, Table S1, Differentially expressed genes in Choice muscle; Table S2, Differentially expressed genes: Choice vs. Standard; Table S3, Differentially expressed genes: Select vs. Standard.
